# Exploring the Impact of Length of Residence and Food Insecurity on Weight Status Among Canadian Immigrants

**DOI:** 10.1177/08901171241246842

**Published:** 2024-04-15

**Authors:** Lei Chai

**Affiliations:** 1 Department of Sociology, 7938 University of Toronto, Toronto, ON, USA

**Keywords:** length of residence, food insecurity, overweight, obesity, immigrants, Canada

## Abstract

**Purpose:**

While the individual impacts of long-term residence and food insecurity on overweight/obesity are well-documented, their combined effect on immigrants’ weight status is less understood. This study examines the interaction between length of residence and food insecurity in predicting overweight/obesity among immigrants and investigates whether this relationship is gender-specific.

**Design:**

A national cross-sectional survey.

**Setting:**

The 2017-2018 Canadian Community Health Survey.

**Subjects:**

Immigrants aged 18 and older (N = 13 680).

**Measures:**

All focal variables were self-reported.

**Analysis:**

Logistic regression models were employed.

**Results:**

Long-term immigrants were more likely to report overweight/obesity than their short-term counterparts (OR = 1.39; *P* < .001). Moreover, immigrants from food-insecure households were at a higher risk of reporting overweight/obesity (OR = 1.27; *P* < .05) compared to those from food-secure households. The analysis further revealed that food insecurity exacerbated the detrimental association between length of residence and overweight/obesity in men (OR = 2.63; *P* < .01) but not in women (OR = .66; *P* > .05).

**Conclusion:**

The findings suggest that long-term immigrant men may be especially susceptible to the compounded chronic stressors of extended residence and food insecurity. Health professionals and policymakers should advocate for psychosocial resources to help mitigate these adverse effects and support the well-being of immigrant populations.

## Purpose

Obesity is a pressing public health concern in Canada, with adult obesity rates having tripled since 1985.^1^ Recognized as a critical risk factor for various chronic health conditions,^2^ obesity is responsible for 61%–74% of type 2 diabetes cases and is associated with 20% of premature Canadian deaths.^3^ Beyond these direct health impacts, obesity also places a considerable economic strain on health care systems, representing nearly 11% of Canada’s total health expenditure.^4^ A breadth of research, including meta-analyses and systematic reviews, has identified numerous risk factors for overweight and obesity in the general population.^5,6^

Despite this extensive evidence, research on predictors of overweight and obesity among immigrants remains limited. Immigrants in Canada typically have a lower reported obesity rate compared to the native-born population (17% vs 30%).^7^ However, analysis by the author of the 2017-2018 CCHS data indicates that long-term residents exhibit a higher incidence of overweight/obesity than their short-term counterparts (53% vs 42%). With foreign-born individuals making up an increasing share of Canada’s population,^8^ understanding the contributing factors influencing overweight/obesity in this group is essential to inform public health prevention and intervention strategies.

Prior research has highlighted that both longer residency^(9)^ and food insecurity^(10)^ independently contribute to an increased risk of overweight and obesity among immigrants, yet the interactive effects between the two have not been extensively explored. This study aims to address this gap by utilizing data from the 2017-2018 Canadian Community Health Survey to investigate the independent and interactive effects of length of residence and food insecurity on immigrant weight status in Canada. This research also explores how these dynamics may differ for men and women. The review of literature and hypotheses will be presented next.

Immigrants generally exhibit better health outcomes than the native-born population, a phenomenon supported by extensive research.^11,12^ This health advantage of immigrants has prompted several explanatory hypotheses. The “healthy immigrant effect” suggests that immigrants may initially be healthier; the “cultural buffering” hypothesis proposes that cultural practices protect against some health risks; and the “salmon bias” hypothesis contends that unhealthy immigrants may return to their country of origin.^9,12,13^ These hypotheses are further developed in studies demonstrating that health disparities evolve over time and are affected by the duration of residence in the host country.^14^

Research increasingly recognizes a pattern of declining health among immigrants with prolonged residency in their new country.^12^ Studies have linked an extended length of stay to a deterioration in health outcomes.^13^ This decline is often attributed to “unhealthy assimilation,”^13^ a phenomenon where immigrants adopting local lifestyle habits experience negative health impacts, such as a shift towards a less active lifestyle or changes in diet. Furthermore, “cumulative stress,”^15^ another significant factor, refers to the extended exposure to chronic stressors. These stressors, including financial strain, cultural adjustment difficulties, and experiences of discrimination, collectively contribute to a decline in immigrants’ mental health.

Concurrently, there is growing interest in how the length of residence impacts health behaviors, particularly overweight and obesity. Some studies reveal that longer residence increases the risk of overweight and obesity among certain immigrant groups,^9^ with variations across different ethnicities.^16^ Other studies, such as those by Cofie and Cuevas,^17^ find a correlation between longer residence and rising obesity risk but are limited by their sample compositions and potential confounding variables. Jung and colleagues^18^ identified a similar pattern, but their study’s limited sample size restricts its generalizability. To overcome these limitations, it is essential to use comprehensive surveys that capture a wide array of ethnic/racial groups and include a complete set of control variables. Theoretical and empirical evidence supports the hypothesis that long-term immigrants are more likely to report overweight/obesity than their short-term counterparts (**Hypothesis 1**).

Food insecurity, defined as “the inability to acquire or consume an adequate diet quality or sufficient quantity of food in socially acceptable ways, or the uncertainty that one will be able to do so,”^19^ is a critical factor that may influence immigrants’ weight status. It is classified as a chronic stressor^20-22^ within the stress process model^23^ and can lead to health challenges by limiting access to coping resources. Recent studies, such as that by Dou and colleagues,^24^ have linked food insecurity to poorer mental well-being among immigrants. Furthermore, Smith and Coleman-Jensen^10^ found that food insecurity was associated with an increased risk of obesity among immigrant adults, possibly due to the prevalence of energy-dense, nutrient-poor diets in food-insecure households.^25^ This line of research informs the hypothesis that immigrants in food-insecure households are more likely to report overweight/obesity than those in food-secure households (**Hypothesis 2**).

Given these individual risk factors, it is important to consider their potential interplay. This study aims to investigate the intersection of length of residence and food insecurity on immigrant weight status, drawing on the stress amplification perspective for theoretical insight.^23^ This perspective posits that stressors often do not act in isolation but rather in combination, exacerbating the negative effects on health. In the context of immigrant health, this could mean that long-term immigrants, who are already dealing with various stressors, may experience an even higher risk of overweight/obesity if they also face food insecurity simultaneously. Although this specific interaction has not been examined, the stress amplification perspective implies that food insecurity could worsen the adverse effects of prolonged residence on weight status (**Hypothesis 3**).

In addition to the combined effects of length of residence and food insecurity on immigrant weight status, this study also considers the role of gender as a potential moderator in this relationship from two sociological frameworks. The “doing gender”^26^ perspective contends that traditional societal norms often position men as the primary breadwinners, viewing their contribution to household finances as fundamental to their masculine identity. When faced with food insecurity—a clear indicator of economic strain—men may interpret this as a personal failure, potentially undermining their masculine identity. This perceived failure could escalate emotional stress and obesity-promoting behaviors such as substance use.^27,28^

Similarly, the gender role socialization perspective^29^ notes that ingrained interpersonal skills typically provide women with broader social networks than men. These networks can offer diverse coping strategies during challenging times. Conversely, cultural norms that reinforce traditional views of masculinity may discourage men from seeking external social support,^30^ limiting their coping mechanisms. For instance, men may be hesitant to share their struggles with food insecurity, whereas women, with their expansive support systems, could access various coping strategies. This disparity in social support access suggests that the negative influence of food insecurity on the relationship between length of residence and overweight/obesity may differ by gender. Both perspectives support the rationale that the moderating role of food insecurity may have a more substantial negative impact on the weight status of male immigrants compared to female immigrants (**Hypothesis 4**). This line of inquiry underscores the need for gender-specific interventions in addressing the health of immigrants in Canada.

## Methods

### Design

The present study utilized the 2017-2018 public use microdata files from the Canadian Community Health Survey (CCHS), administered by Statistics Canada.^31^ The CCHS is a nationally representative survey focusing on the health and well-being of Canadians aged 12 and older. The survey excludes full-time members of the Canadian Forces, residents of the Territories, those on reserves, and individuals in some remote areas or institutions. Data for the survey were collected through both telephone and personal interviews. For further details about the sample design and data collection procedures, readers are referred to Statistics Canada.^31^ The original sample consisted of 113 290 individuals, with an overall response rate of 60.8%. The present study used a publicly available microdata file of the 2017-2018 CCHS, which does not require ethics board approval.

### Sample

Sampling weights were applied to all analyses to ensure the sample was representative of the larger population. For this study, the analyses were restricted to immigrants aged 18 and older. Missing values were minimal, ranging from .12% (ie, household size) to 8.04% (ie, weight status). Listwise deletion was used for handling missing values, resulting in an analytical sample of 13 680 individuals.

### Measures

*Length of residence in Canada since migration* was coded as either “0-9 years” or “10 or more years.”^13,15^

*Weight status* was coded as “underweight,” “normal weight,” “overweight/pre-obesity,” “obesity class I,” “obesity class II,” and “obesity class III.” This classification, adopted by the CCHS from Health Canada and the World Health Organization (WHO),^32,33^ was dummy-coded, with 1 indicating “overweight/obesity class I/II/III” and 0 indicating “underweight/normal weight.”

*Food insecurity* was assessed using 18 items, originally developed and validated by the US Department of Agriculture and later adapted for the CCHS by Health Canada.^34^ The responses were coded as “food secure,” “moderately food insecure,” and “severely food insecure.” For analysis, these latter two groups were combined into one category to ensure adequate cell size, with 1 indicating “moderately/severely food insecure” and 0 indicating “food secure.”^22,35^

*Gender* was coded as “men” and “women.”

A set of sociodemographic characteristics was included as control variables to account for their potential influence on the focal associations. *Age group* was recoded as “18 to 34,” “35 to 54,” and “55 and older.” *Race* was categorized as “White” and “non-White.” As per Statistics Canada, the “non-White” category includes South Asian (eg, East Indian, Pakistani, Sri Lankan), Chinese, Black, Filipino, Latin American, Arab, Southeast Asian (eg, Vietnamese, Cambodian, Malaysian, Laotian), West Asian (eg, Iranian, Afghan), Korean, Japanese, and other specified ancestries. These categories were grouped as a form of disclosure control.^31^ *Household size* was treated as a continuous variable. *Marital status* was recoded as “married,” “common-law,” “previously married,” and “single/never married.” *Education* was coded as “less than secondary school graduation,” “secondary school graduation without post-secondary education,” and “post-secondary certificate, diploma, or university degree.” *Main activity* was coded as “working or on vacation (from paid work),” “looking for paid work,” “going to school (including vacation from school),” “retired,” “long-term illness,” and “other.” The “other” category was grouped as a form of disclosure control.^31^ *Total household income* was coded as “no income or less than $20,000,” “$20,000 to $39,999,” “$40,000 to $59,999,” “$60,000 to $79,999,” and “$80,000 or more.” *Region* was coded as “Atlantic,” “Central,” “Prairie,” “West,” and “Northern territories.”

### Analysis

Logistic regression models were conducted. In Table 2, Model 1 examined the focal associations of length of residence and food insecurity with weight status without control variables, while Model 2 examined them with a full set of control variables. Model 3 assessed the two-way interaction to investigate whether the association between length of residence and weight status varied by food insecurity. Model 4 introduced the three-way interaction term to examine gender differences in the moderating influence of food insecurity on the relationship between length of residence and weight status. Table 3 further unpacked the three-way interaction, documenting the two-way interaction for men (Model 1) and women (Model 2) separately, each with a full set of control variables. All analyses were conducted using Stata/SE 14.2.

## Results

Table 1 presents descriptive statistics for all study variables. Three key patterns emerged. First, the majority of respondents in the study sample were long-term immigrants (73.51%), with a similar distribution among men (74.27%) and women (72.76%). Second, while the full sample showed an approximately even distribution of weight status, a higher percentage of men (56.44%) reported being overweight/obese compared to women (43.58%). Third, less than 10% of respondents indicated food insecurity, with comparable rates for men (7.39%) and women (9.61%).

**Table 1.** Descriptive Statistics for all Study Variables.

	Full sample		Men		Women	
	M/%	SE	M/%	SE	M/%	SE
Length of residence						
0-9 years (ref.)	26.49		25.73		27.24	
10 or more years	73.51		74.27		72.76	
Weight status						
Normal weight/underweight (ref.)	50.07		43.56		56.42	
Overweight/obesity	49.93		56.44		43.58	
Food insecurity						
No (ref.)	91.49		92.61		90.39	
Yes	8.51		7.39		9.61	
Age group						
18-34 (ref.)	23.24		23.77		22.73	
35-54	40.49		40.03		40.93	
55 and older	36.27		36.20		36.34	
Race						
White (ref.)	32.70		31.50		33.87	
Non-white	67.30		68.50		66.13	
Household size	3.04	.02	3.07	.03	3.00	.03
Marital status						
Married (ref.)	65.61		68.29		63.01	
Common-law	5.20		5.46		4.95	
Previously married	11.31		7.12		15.39	
Single/never married	17.87		19.12		16.65	
Education						
Less than secondary (ref.)	8.03		7.31		8.74	
Secondary	20.42		19.73		21.09	
Post-secondary	71.55		72.95		70.17	
Main activity						
Working (ref.)	60.94		68.57		53.49	
Looking for paid work	2.44		2.99		1.90	
Going to school	5.57		5.18		5.94	
Retired	19.18		18.43		19.91	
Long-term illness	1.51		1.55		1.48	
Other	10.37		3.28		17.28	
Household income						
Less than $20,000 (ref.)	6.84		6.41		7.25	
$20,000 to $39,999	13.64		11.52		15.71	
$40,000 to $59,999	15.38		14.88		15.88	
$60,000 to $79,999	12.62		13.16		12.10	
$80,000 or more	51.52		54.03		49.06	
Region						
Atlantic (ref.)	.94		.98		.91	
Central	65.23		65.31		65.15	
Prairie	16.41		16.05		16.75	
West	17.32		17.56		17.09	
Northern territories	.10		.10		.10	
N	13 680		6396		7284	

Note: All means (M) and proportions (%) are weighted, but unweighted Ns.

Table 2 presents results from a series of logistic regression models. In Model 1, prior to incorporating control variables, it was found that long-term immigrants were more likely to report overweight/obesity than short-term immigrants (OR = 1.53; *P* < .001; 95% CI = 1.34, 1.76), At the same time, food insecurity heightened the risk of overweight/obesity (OR = 1.30; *P* < .01; 95% CI = 1.07, 1.58). In Model 2, after accounting for gender and a full set of control variables, similar patterns persisted for length of residence (OR = 1.39; *P* < .001; 95% CI = 1.19, 1.62) and food insecurity (OR = 1.27; *P* < .05; 95% CI = 1.03, 1.56), supporting **Hypotheses 1 and 2**.

**Table 2.** Logistic Regression results Predicting Overweight/Obesity Risk.

	Model 1: Without control Variables			Model 2: Model 1 + Gender + control variables			Model 3: Model 2 + two-way interaction			Model 4: Model 2 + three-way interaction		
	OR	95% CI	SE	OR	95% CI	SE	OR	95% CI	SE	OR	95% CI	SE
Length of residence												
0-9 years (ref.)												
10 or more years	1.53***	[1.34, 1.76]	(.11)	1.39***	[1.19, 1.62]	(.11)	1.36***	[1.15, 1.61]	(.11)	1.60***	[1.26, 2.03]	(.19)
Food insecurity												
No (ref.)												
Yes	1.30**	[1.07, 1.58]	(.13)	1.27*	[1.03, 1.56]	(.13)	1.13	[.80, 1.61]	(.20)	1.92**	[1.23, 3.02]	(.44)
Length of residence X food insecurity												
10 or more years X yes							1.20	[.78, 1.84]	(.26)	.65	[.38, 1.12]	(.18)
Gender												
Women (ref.)												
Men				1.79***	[1.58, 2.02]	(.11)	1.79***	[1.58, 2.02]	(.11)	2.33***	[1.78, 3.04]	(.32)
Length of residence X gender												
10 or more years X men										.73*	[.54, .97]	(.11)
Food insecurity X gender												
Yes X men										.31***	[.16, .61]	(.11)
Length of residence X food insecurity X gender												
10 or more years X yes X men										3.90**	[1.68, 9.05]	(1.68)
Intercept	.71			1.26			1.28			1.11		
N	13 680			13 680			13 680			13 680		

Note: A full set of control variables includes age group, race, household size, marital status, education, main activity, household income, and region.

OR = odds ratio. SE = standard error. Models include sample survey weights. ***P < .001; **P < .01; *P < .05.

**Figure 1.**
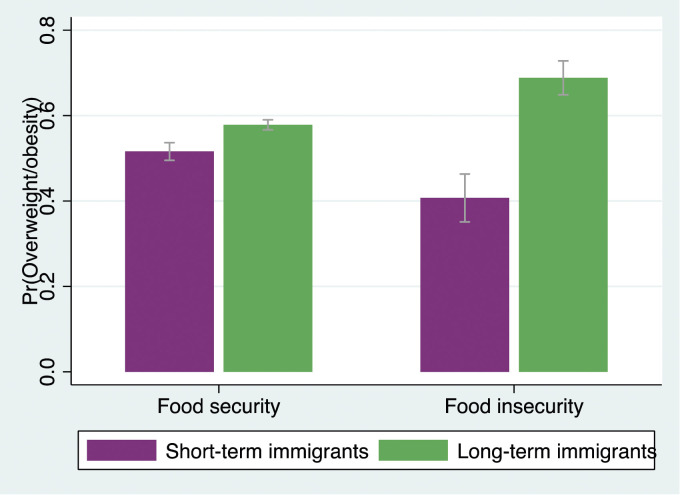
Predicted probabilities of reporting overweight/obesity by length of residence and food insecurity among men.

**Figure 2.**
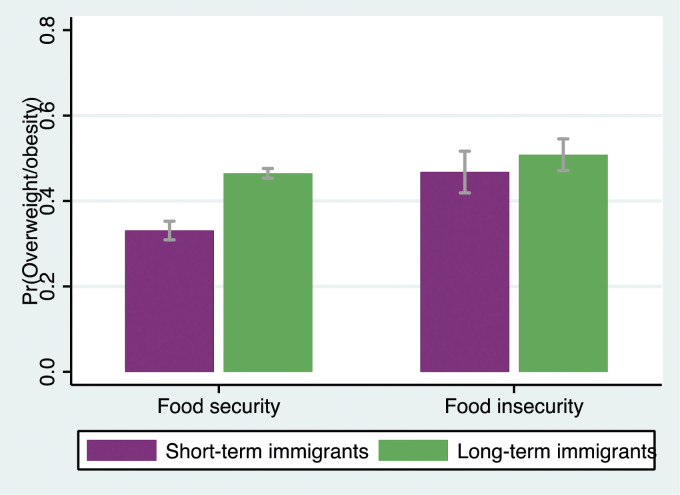
Predicted probabilities of reporting overweight/obesity by length of residence and food insecurity among women.

Model 3 tested the interaction between length of residence and food insecurity and revealed that food insecurity did not moderate the association between length of residence and weight status (OR = 1.20; *P* > .05; 95% CI = .78, 1.84), thus not supporting **Hypothesis 3**. However, Model 4 demonstrated that the three-way interaction term, which accounts for gender differences (ie, length of residence X food insecurity X gender), was significant (OR = 3.90; *P* < .01; 95% CI = 1.68, 9.05). To further explore this three-way interaction, the two-way interaction terms (ie, length of residence X food insecurity) for men and women were separately presented in Table 3. Model 1 suggested that among men, food insecurity amplified the adverse association between length of residence and overweight/obesity (OR = 2.63; *P* < .01; 95% CI = 1.38, 4.99). Conversely, Model 2 revealed that for immigrant women, food insecurity did not serve as a moderating factor (OR = .66; *P* > .05; 95% CI = .38, 1.14). Additional analyses were conducted based on recent recommendations for testing interaction effects with a non-linear outcome variable (results not shown but available upon request).^36^ The same patterns were observed. Figures 1 and 2 depict these two-way interactions, showing the predicted probabilities of reporting overweight/obesity based on length of residence and food insecurity for both genders.^36^ The results provided partial support for **Hypothesis 4**.

**Table 3.** Logistic Regression results Predicting Overweight/Obesity Risk.

	Model 1: Men			Model 2: Women		
	OR	95% CI	SE	OR	95% CI	SE
Length of residence						
0-9 years (ref.)						
10 or more years	1.23	[.98, 1.54]	(.14)	1.59***	[1.24, 2.04]	(.20)
Food insecurity						
No (ref.)						
Yes	.63	[.37, 1.05]	(.17)	1.83*	[1.16, 2.89]	(.43)
Length of residence X food insecurity						
10 or more years X yes	2.63**	[1.38, 4.99]	(.86)	.66	[.38, 1.14]	(.18)
Intercept	1.63			1.40		
N	6396			7284		

Note: All models include a full set of control variables: age group, race, household size, marital status, education, main activity, household income, and region. OR = odds ratio. SE = standard error. Models include sample survey weights. ***P < .001; **P < .01; *P < .05.

## Discussion

The “unhealthy assimilation”^9^ hypothesis posits that long-term immigrants may adopt unhealthy lifestyles as their length of residence increases, potentially leading to deteriorated physical health outcomes. Guided by this hypothesis, public health scholars have investigated similar patterns in health behaviors, such as overweight/obesity.^9^ Consistent with prior research in the United States,^9^ this study revealed that long-term immigrants were more likely than short-term ones to report overweight/obesity. While the aforementioned findings highlight a notable trend in the weight status of long-term immigrants, it is important to consider other influencing factors. For instance, the relationship between length of residence and weight status may be influenced by age at migration.^9^ For instance, Afable and colleagues^9^ found that among Filipino immigrants in New York, the positive association between length of residence and being overweight was significant only for immigrants younger than 31 years. Given that the CCHS does not capture age at migration, future research should consider exploring its potential moderating effect on the relationship between length of residence and weight status, utilizing nationally representative samples of immigrants.

Concurrently, the analysis found that food insecurity elevated the risk of overweight/obesity, aligning with the stress process model’s predictions.^23^ This finding is particularly notable as research on the relationship between food insecurity and weight status is predominantly conducted in the general population or among children and adolescents.^37^ While evidence suggests that food insecurity increases the risk of obesity among American immigrant adults,^10^ this research’s exploration of this dynamic in Canada contributes new insights to the literature on food insecurity and immigrant weight status.

Furthermore, this study examined the interaction between length of residence and food insecurity in relation to immigrant weight status. Adopting the stress amplification viewpoint,^23^ it was probed whether the detrimental association between length of residence and overweight/obesity was more pronounced for individuals facing food insecurity. Contrary to expectations, the results did not support this hypothesis. However, the introduction of gender into the analysis added complexity, suggesting that the moderating role of food insecurity may be contingent upon gender. The analysis of the three-way interaction concerning gender differences was statistically significant. Specifically, the two-way interaction by gender showed that food insecurity intensified the adverse association between length of residence and overweight/obesity in men, but not in women.

These insights align with predictions from the “doing gender”^26^ and gender socialization^29^ perspectives. For example, considering the role of the breadwinner often associated with heterosexual men’s masculinity, food insecurity may intensify emotional stress in men, potentially leading to obesity-promoting behaviors such as substance use.^27,28^ Thus, the adverse moderating effect of food insecurity may be more pronounced for men. Concurrently, cultural norms often expect men to sustain independence during personal struggles, while women may seek diverse sources of social support. Thus, the adverse moderating role of food insecurity could be intensified for men due to their relative lack of social support. While these propositions have theoretically merit, they are speculative and warrant further examination in future research. Qualitative inquiries could provide a richer, context-orientated understanding of this matter.

This study has several limitations. First, due to the cross-sectional design of the CCHS, causal relationships between length of residence, food insecurity, and immigrant weight status cannot be determined. Longitudinal studies are required to delve deeper into potential causal effects. Second, evidence suggests that the relationships between length of residence, food insecurity, and weight status may vary based on race/ethnicity.^16,17^ However, as previously mentioned, the public use microdata from the CCHS lack detailed racial/ethnic categorizations, which precludes an examination of potential racial/ethnic differences.

Third, all focal measures are based on self-reporting, potentially introducing recall bias and affecting the accuracy of the results. Still, it is worth noting that self-reported measures are common in nationally representative surveys. While clinical obesity measures, such as dual-energy X-ray absorptiometry, may offer more accurate results, their data collection can be both time-intensive and costly, possibly leading to nonrepresentative samples. Fourth, the study’s categorization of length of residence may raise concerns among some readers. In the U.S., the classification of length of residence varies.^12,27^ In Canada, the distinctions typically contrast long-term vs short-term immigrants.^13,15^ Future studies need to further explore the influence of residence duration on weight status, especially as some evidence suggests that those with a middle-length of residence, as opposed to long-term ones, may report more health challenges than their short-term counterparts.^12^

In spite of these limitations, this study offers multiple strengths. It not only sheds light on the independent associations of length of residence and food insecurity with immigrant weight status using a nationally representative cross-sectional survey but also stands outs for its exploration of the gendered moderating effects of food insecurity. Such an exploration implies that solely viewing length of residence and food insecurity as independent predictors may mask the full picture of overweight/obesity disparities among immigrants. Future research should delve into possibly psychosocial resources that could offset the adverse moderating effect of food insecurity. Social support, known for its significant role in the stress process model,^23^ emerges as a potential resource. Social support has consistently shown a positive association with health and well-being.^21^ Numerous studies have found that elevated levels of social support can act as buffers against the adverse association between stressors and health.^23^ In conclusion, these results underscore the need for interventions aimed at enhancing psychosocial resources, like social support, among immigrants—particularly men—to possibly mitigate the challenging moderating effects of food insecurity on the relationship between length of residence and weight status.

**So What?**

**What is Already Know on this Topic?**

The individual impacts of long-term residence and food insecurity on overweight/obesity have been established.

**What does this Article Add?**

Less is known about their combined effect on the weight status of immigrants. This study aims to fill this gap by examining the interaction between length of residence and food insecurity in predicting overweight/obesity among immigrants and investigates whether this relationship differs by gender.

**What are the Implications for Health Promotion Practice or Research?**

Long-term immigrants were more likely to report overweight/obesity than their short-term counterparts. Moreover, immigrants living in food-insecure households had a higher risk of reporting overweight/obesity than those living in food-secure households. The analysis also revealed that food insecurity amplified the adverse association between length of residence and overweight/obesity for men but not for women. The results suggest that long-term immigrant men may be particularly vulnerable to the combined chronic stressors of prolonged residence and food insecurity. Health professionals and policymakers are encouraged to advocate for psychosocial resources that could potentially help mitigate these detrimental effects and support the well-being of immigrant populations.
